# Targeting procaspase-3 with WF-208, a novel PAC-1 derivative, causes selective cancer cell apoptosis

**DOI:** 10.1111/jcmm.12566

**Published:** 2015-03-08

**Authors:** Fangyang Wang, Yajing Liu, Lihui Wang, Jingyu Yang, Yanfang Zhao, Nannan Wang, Qi Cao, Ping Gong, Chunfu Wu

**Affiliations:** a Department of Pharmacology, Shenyang Pharmaceutical UniversityShenyang, China; b Department of Medicinal Chemistry, Shenyang Pharmaceutical UniversityShenyang, China

**Keywords:** procaspase-3 activator, anticancer, new derivative, PAC-1

## Abstract

Caspase-3 is a critical effector caspase in apoptosis cascade, and is often over-expressed in many cancer tissues. The first synthesized procaspase-3 activator, PAC-1, induces cancer cell apoptosis and exhibits antitumour activity in murine xenograft models. To identify more potent procaspase-3 activators, a series of compounds were designed, synthesized and evaluated for their ability of inducing cancer cell death in culture. Among these compounds, WF-208 stood out by its high cytotoxicity against procaspase-3 overexpressed HL-60 cells. Compared with PAC-1, WF-208 showed higher cytotoxicity in cancer cells and lower toxicity in normal cells. The further investigation described herein showed that WF-208 activated procaspase-3, degraded IAPs (The Inhibitors of apoptosis proteins) and leaded to caspase-3-dependent cell death in tumour cells, which possibly because of the zinc-chelating properties. WF-208 also showed greater antitumour activity than PAC-1 in murine xenograft model. In conclusion, we have discovered WF-208 as a promising procaspase-3 activating compound, with higher activity and higher cell selectivity than PAC-1.

## Introduction

A critical event in the apoptotic cascade is the proteolytic activation of procaspase to active caspase. The most common and important effector caspase is caspase-3, which exists as a zymogen and activated by initiator caspases, mainly caspase-8 and caspase-9. The activated caspase-3 cleaves more than 300 substrates, leading to apoptosis 1–3. Accumulating evidence has revealed that procaspase-3 is over-expressed in various human tumours, including colon cancer 4, lung cancer 5, melanoma 6, hepatoma 7, breast cancer 8, lymphoma 9 and neuroblastoma 10. But owing to the mutation or aberrant expression of an assortment of proteins in the apoptotic cascade in cancerous cells, procaspase-3 may not be successfully activated to caspase-3 in cancer cells. Cancerous cells typically possess a lower sensitivity than normal cells to pro-apoptotic signals, such as endogenous signal and some chemotherapeutic agents 11. Thus, the activation of procaspse-3 to caspase-3 is a valuable approach to the reactivation of apoptotic cascades as an anticancer strategy 12.

In previous study, the first procaspase-3 activator, PAC-1 (discovered in 2006) enhances procaspase-3 activity *in vitro*, induces death of cancer cells in culture, and is effective in mouse xenograft models 13. PAC-1 activates procaspase-3 *in vitro* through the chelation of inhibitory zinc ions. The structure-activity relationship (SAR) analysis indicated that the metal chelating properties of ortho-hydroxy *N*-acylhydrazone is necessary for zinc binding and procaspase-3 activation 14. In addition, PAC-1 also has desirable pharmacokinetic properties 15. However, further reports have suggested that PAC-1 exhibit neurotoxicity *in vitro* and *in vivo* 16,17.

To further develop better procaspase-3-activating compounds, we synthesized a series of novel 1,2,4-oxadiazole substituted hydrazide derivatives and evaluated their ability to induce death of cancer cells in culture. Among these compounds, WF-208 and WF-210 had the highest potency. In our previous report, we have described the comprehensive investigation of the mechanisms that underlie the activity of WF-210 18. In present article, we report herein the structure-function relationship of these derivatives and further investigated for its antitumour activity and its mechanism of WF-208 *in vitro* and *in vivo*.

## Materials and methods

### General procedure for the synthesis of PAC-1 analogues

PAC-1 was synthesized as described previously 13. The preparation of the target compounds is outlined in scheme8. The commercially available 4-(chloromethyl) benzonitrile reacted with substituted phenol 1 in DMF to obtain 4-(substituted phenoxymethyl) benzonitrile 2, which was treated with hydroxylamine hydrochloride to get compound 3. Cyclization of 3 with 2-chloroacetyl chloride in toluene readily afforded 1,2,4-oxadiazole derivative 4, which was nucleophilic substituted with piperazine in ethanol to give intermediate 5. Reaction of 5 with ethyl chloroacetate afforded 6, which was followed by hydrazine hydrate in EtOH to get acylhydrazines 7. Acylhydrazines 7 were condensed with corresponding benzaldehydes 8, 10 or 13, respectively to obtain the target compounds A_1_–A_4_, B_1_–B_12_ and C_1_–C_2_. The detailed procedure is illustrated in Supplementary materials and methods.

**Figure 8:**
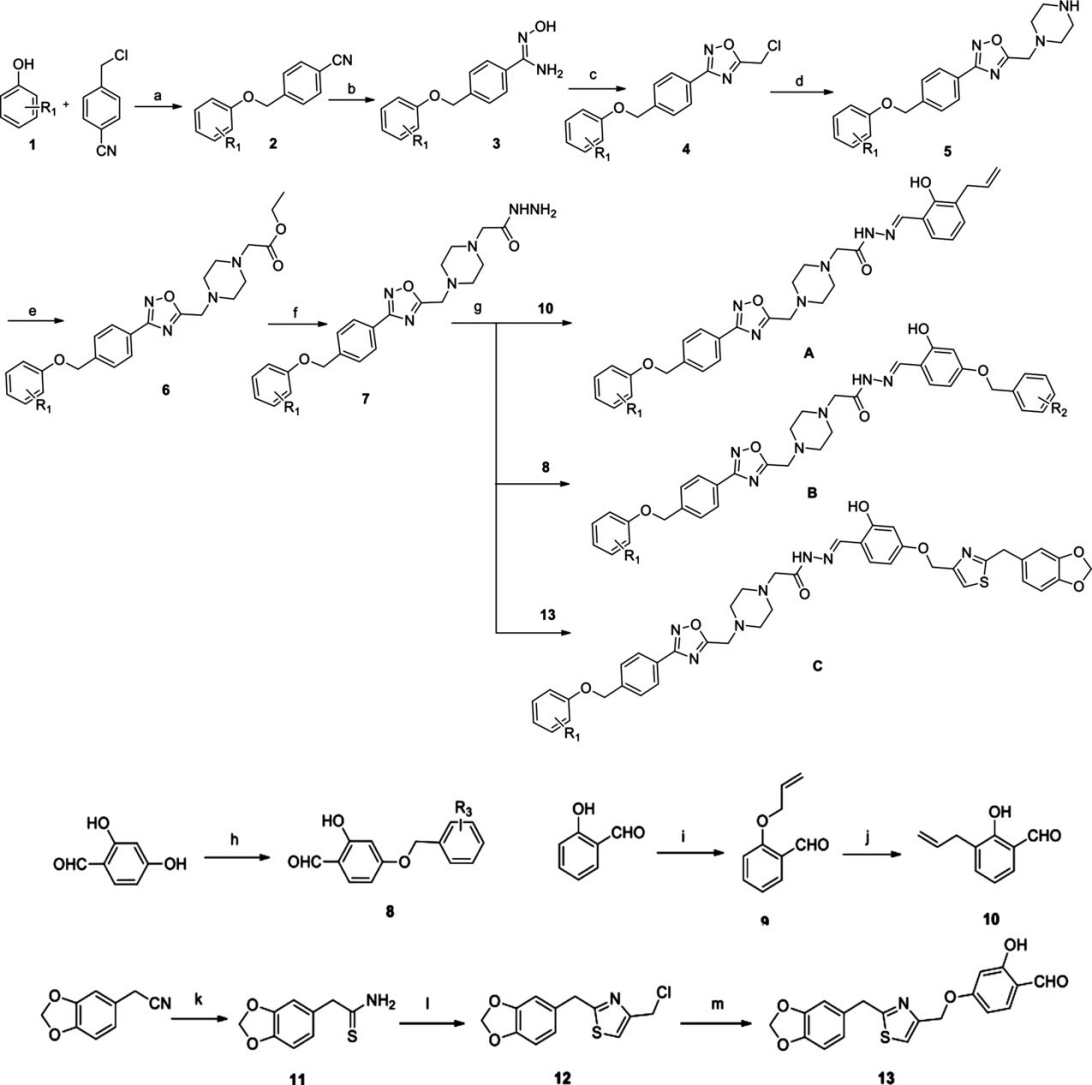
Scheme The preparation of the target compounds.

### Cell lines and cell culture

Human malignant cell lines, A549, Colo-205, DU145, NCI-H226, Hep-3B, Hep-G2, K562, MCF-7, U-87, GBC-SD, MDA-MB-435S, PC-3, U-937, HL60, SH-SY5Y and human normal cell lines, MCF-10A and L-02, HUVEC were obtained from the American Type Culture Collection (Manassas, VA, USA) and National Center for Medical Culture Collection (Shanghai, China). They were routinely cultured in RPMI 1640, DMEM or DMEM/F12 (Gibco, New York, USA) supplemented with 10% foetal bovine serum (Gibco, New York, USA) and maintained at 37°C in a humidified incubator with 5% CO_2_.

Human peripheral blood lymphocytes were obtained as described previously 19. In brief, after diluting blood with PBS, lymphocytes were isolated by centrifugation over a density gradient of lymphocytes extracting solution for 15 min. at 280 × g. The cells were harvested, washed with PBS twice, and suspended in complete RPMI 1640 (Gibco, New York, USA) with 10% foetal bovine serum (Gibco, New York, USA).

### Cell viability assay

The cell viability effects of the tested compounds were determined by MTT assay. Cells (1 × 10^4^ cells/100 μl/well) were seeded into 96-well culture plates. After overnight incubation, cells were treated with various concentrations of compounds for 72 hrs. Subsequently, 10 μl MTT solution (2.5 mg/ml in PBS) was added to each well, than the cells were incubated for an additional 3–4 hrs at 37°C. After centrifugation (200 × g, 10 min.), the medium with MTT was aspirated, followed by the addition of 100 μl DMSO. The optical density of each well was measured at 570 nm with a Biotek Synergy TM HT Reader.

### Caspase-3 activation assay

Procaspase-3 protein was purchased from R&D System (Minneapolis, USA). Procaspase-3 protein was added into 384-well plates. Tested compounds (or DMSO as control) were then added to achieve a final concentration of procaspase-3 of 50 ng/ml. Each plate was incubated for 2 hrs at 37°C. Then caspase-3 peptide substrate (acetyl Asp-Glu-Val-Asp-AFC (Ac-DEVD-pNa), 200 μM) were added. The absorbance of each well was measured at 405 nm in kinetic mode for 10 min 13.

### Assay of zinc inhibition of procaspase-3 activation

HEPES buffer [with 50 mM HEPES and 300 mM NaCl (pH 7.4)] was treated with Chelex resin to remove trace bivalent cations (including zinc ion). ZnSO_4_ and different concentration PAC-1 or WF-208 was added to procaspase-3 in HEPES to yield a final concentration of 0.5 μM procaspase-3. After 12 hrs of incubation at 37°C, Ac-DEVD-pNA in buffer [50 mM Hepes (pH 7.4), 100 mM NaCl, 10 mM DTT, 0.1 mM EDTA disodium salt, 0.10% Chaps, 10% glycerol] was added. The final concentration Ac-DEVD-pNA is 0.4 μM. Then the plate was read absorbance at 405 nm in kinetic mode for 10 min. On the other hand, after various time of incubation at 37°C, western blotting was used to detect the cleavage of procaspase-3 13,14.

### Annexin V/PI staining assay

Compounds were incubated with HL-60 and U-937 cells for 24 hrs. The cells were harvested and double-stained with FITC-Annexin V Apoptosis Detection Kit (BD Biosciences, San Jase, USA). Cells that positively stained with Annexin V and negatively stained with PI were considered to be early apoptotic.

### Immunofluorescence

HL-60 cells were treated with the 50 μM of PAC-1 and 10 μM WF-208 for 0.5, 1, 3, 6, 12 and 24 hrs. After such treatment, cells were fixed in 4% paraformaldehyde for 15 min., washed with PBS. Subsequently, cells were blocked with 10% bovine serum albumin (BSA) and Triton X-100 for 1 hr at room temperature and incubated at 4°C overnight with anti-cleaved caspase-3 rabbit mAb (1:100) diluted in 1% BSA. After three times of washing with PBS, cells were incubated with secondary antibody (FITC-conjugated goat anti-rabbit IgG, 1:200) for 1 hr at room temperature. Hoechst 33342 was used as a double staining for the nucleus. After washing with PBS, digital images of the cells captured under with ImageXpress 5000 (Molecular Devices, Sunnyvale, CA, USA). The images were quantified and analysed by using MetaXpress software (Molecular Devices). Cleaved caspase-3 level was expressed as the percentage of positively FITC-stained cells.

### Transient transfection

HL-60 cells were transfected with procaspase-3 siRNA (Invitrogen, New York, USA, 100 nmol/l final concentration, Sense siRNA Sequence: GUCUAACUGGAAAACCCAAtt Antisense siRNA Sequence: UUGGGUUUUCCAGUUAGACtt) by Lipofectamine 2000 (Invitrogen) according to the manufacturer’s instructions. Scrambled siRNA (Invitrogen) was control. The cDNA of human procaspase-3 was cloned into pIRES2-EGFP expression vector, and procaspase-3 and EGFP genes were separately expressed. The pIRES2-EGFP and pIRES2-EGFP-procaspase-3 were transiently transfected into MCF-7 cells. Expression of procaspase-3 was verified by western blotting.

### Western blot

Approximately 1 × 10^7^ HL-60 cells were gathered after pre-treatment with PAC-1 and WF-208 as previously described. The tumour tissue protein was purified according to the reported methods 13. Western blotting was performed as previously described 20. All antibodies were purchased from Cell Signaling Technology. In brief, an equal amount of total protein extracts from cultured cells or tissues were fractionated by 10–15% SDS-PAGE and electrically transferred onto polyvinylidene difluoride (PVDF) membranes. Different primary antibodies and corresponding horseradish peroxidase-conjugated secondary antibodies were used to detect the proteins. The bound secondary antibodies on the PVDF membrane were reacted with ECL™ Western Blotting Reagents detection reagents (Thermo Fisher, Rockford, USA) and exposed to X-ray films. Results were normalized to the internal control β-actin.

### Quantitative PCR

About 1 × 10^6^ cells were gathered after pre-treatment with PAC-1 or WF-208 as described previously. Total RNA was isolated using RNeasy Mini Kit (Qiagen, Duesseldorf, Germany) as described by the product’s instruction. One microgram of RNA was reversely transcribed with ReverAid First Stand cDNA Sythesis Kit (Thermo Fisher, Rockford, USA). For quantitative PCR, analysis was carried out by using iQ SYBR Green Supermix (Bio-Rad, West Berkeley, USA) and the CFX96 RT-PCR Detection System (Bio-Rad) as instructed by the manufacturer. Primers were GAPDH reverse primer 5′-CCCTCAACGACCACTTTGTCA-3′ and forward primer 5′-TTGCCGACAGGATGCAGAA-3′; XIAP reverse primer 5′-TTGCCGACAGGATGCAGAA-3′ and forward primer 5′-GCCGATCCACACGGAGTACT-3′; Survivin reverse primer 5′-GGAAACTGCGGAGAAAGTG-3′ and forward primer 5′-TAAACCCTGGAAGTGGTGC-3′. The PCR cycle started with 5 min. at 95°C, followed by 35 cycles of 3 steps (30 sec. at 95°C, 25 sec. at 56°C, 20 sec. at 72°C). The result was calculated as 2^−ΔΔCt^ of real-time fluorescence intensity.

### Human prostatic carcinoma xenograft mouse model

To determine the anti-tumour activity of WF-208 *in vivo*, viable human prostate cancer PC-3 cells (5 × 10^6^/100 μl PBS per mouse) were subcutaneously (s.c.) injected into the right flank of 7- to 8-week old male SCID mice. When the average tumour volume reached 100 mm^3^, mice were randomly divided into various treatment and control group (eight mice per group). Tumour size was measured once every 2 days with a calliper (calculated volume = shortest diameter^2^ × longest diameter/2). Body weight, diet consumption and tumour size were recorded once every 2 days. After 2 weeks, mice were killed and tumours were excised and stored at −80°C until further analysis.

### Statistical analysis

Data were expressed as mean ± SEM. Differences between experimental groups were evaluated by one-way anova and Tukey’s *post hoc* test using the SPSS11.5 software package for Windows (SPSS, Chicago, IL, USA). Statistical significance was achieved was *P*-value is less than 0.05 (two-tailed test).

## Results

### Design and synthesis of a series of PAC-1 derivatives

**Figure 1:**
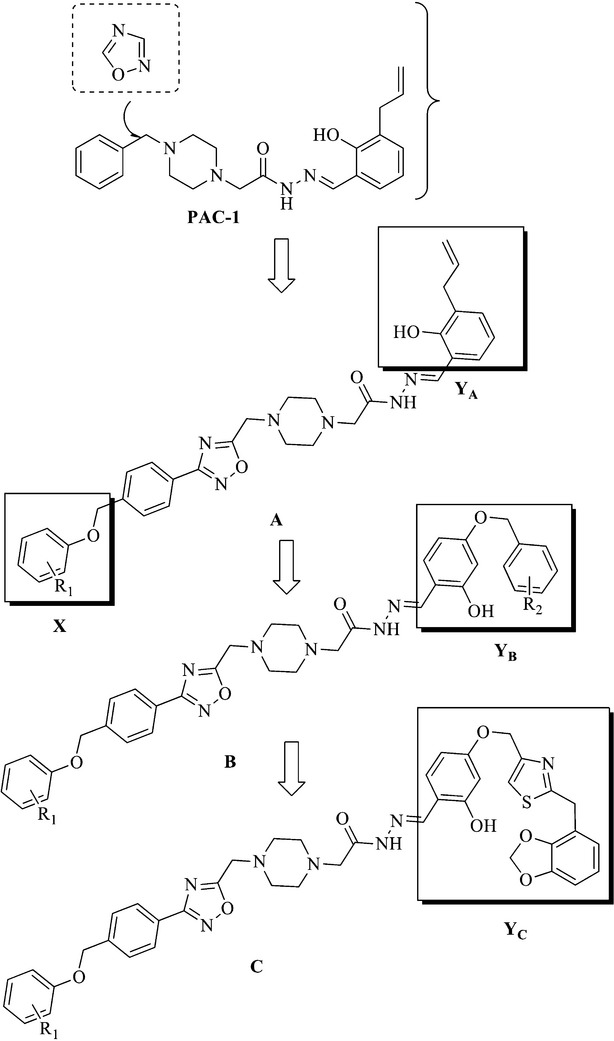
The general structures of target compounds.

Oxadizole, an important heterocyclic ring present in a variety of biologically active molecules, is responsible for effective anticancer treatment. A common type of oxadizole is 1,2,4-oxadizole exerts various physiological effects on organisms 21,22. Hence, in consideration of the good potent of PAC-1, we inserted a 1,2,4-oxadizole between the structures of piperazine and phenyl ring of PAC-1 as a linker to investigate the influence of the altering PAC-1 structure on anticancer effects. Moreover, various substituted phenoxymethyl moiety (X) were introduced into the terminal of PAC-1 to extend the length of carbon chain and to explore the influence of electronic and steric effects on the antitumour activity. Therefore, we designed and synthesized a series of 1,2,4-oxadiazole substituted hydrazide derivatives A (Fig.1). Because of a previous SAR study shown that the PAC-1 derivatives lacking the ortho-hydroxyl group displayed little anticancer activity *in vitro* 23, we retained the substituted 2-hydroxyl phenyl ring moiety and introduced various substituted benzyloxy groups to get compounds B_1_–B_12._ Furthermore, to investigate the effect of a heterocylic ring for moiety Y_B_, we replaced the phenyl group with a 1,3-benzodioxole group, and then introduced a thiazol group as a linker to obtain compounds C_1_ and C_2_ (WF-208) (Fig.1, Table1).

**Table 1:**
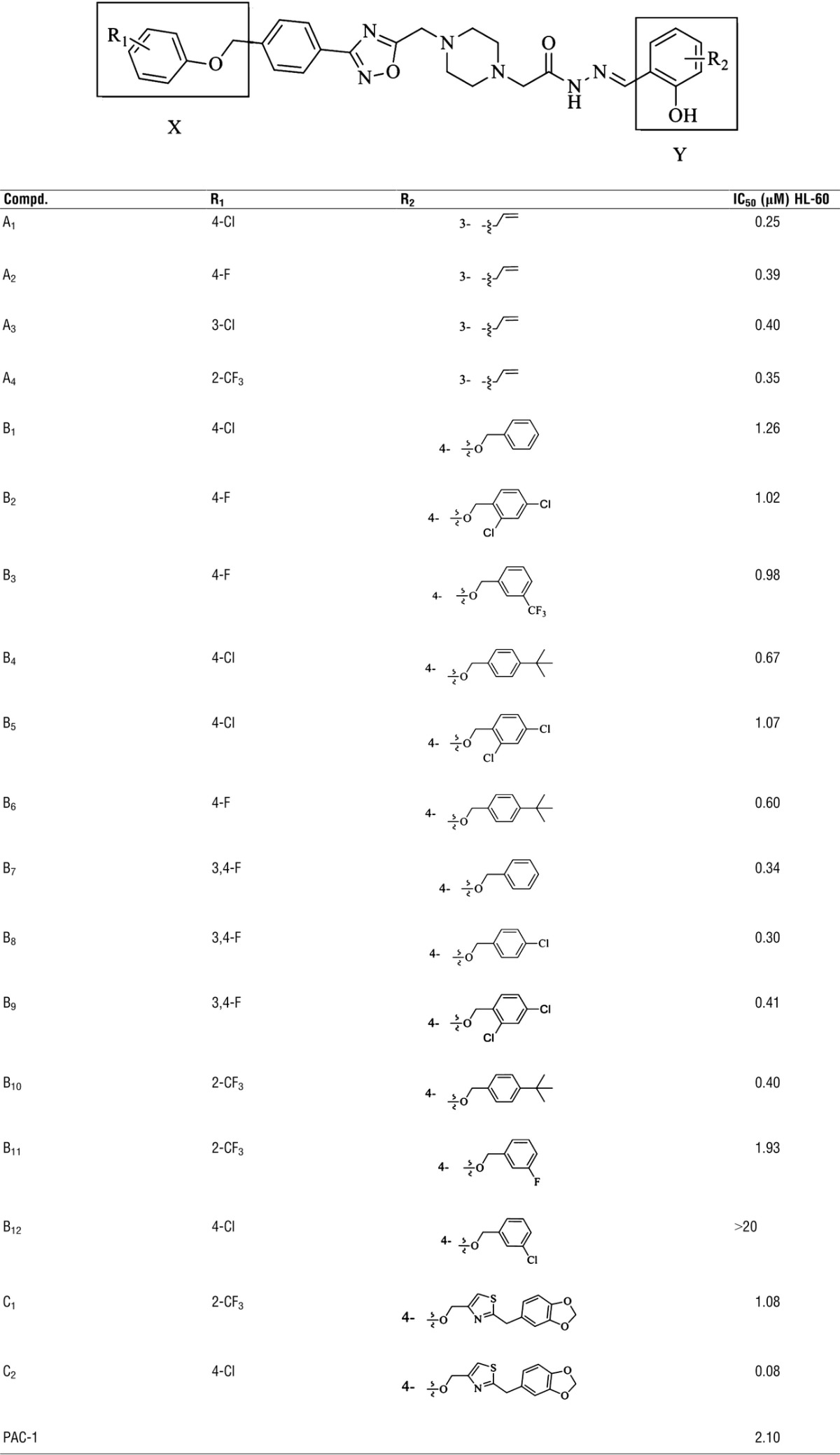
Substituents and cytotoxicity of compounds against HL-60 cell line

### Evaluation of the series of PAC-1 derivatives

The cytotoxic activity of synthesized 1,2,4-oxadiazole substituted hydrazide derivatives was evaluated against human leukaemia cell line HL-60 with procaspase-3 overexpression 13. The results, expressed as IC_50_, were summarized in Table1 with PAC-1 as a reference control. As shown in Table1, most of the compounds exhibited cytotoxicity of HL-60 cells. Generally, compounds with the same part X, but different parts Y shown obvious difference in cytotoxic activities. For instance, compounds A_1_–A_4_ with 3-allyl-2-hydroxyphenyl group and compounds C_1_–C_2_ were more potent than compounds B_1_–B_12_ with 4-(benzyloxy)-2-hydroxyphenyl group. Especially, compound C_2_ (WF-208) displayed excellent antitumour activity against HL-60 cells (IC_50_ = 0.08 μM), which was 26 times lower than PAC-1 (IC_50_ = 2.10 μM), indicating it was the most promising compound. Compounds A_1_–A_4_, with the same part Y but different electron-withdrawing groups (EWGs) on the phenyl of moiety X, showed comparable inhibitory activities against HL-60, suggested that these substituent groups on moiety X exerted little influence on the activity. In the series of compounds B, compared compounds B_4_ with B_1_ and B_5_, B_6_ with B_2_ and B_3_, B_10_ with B_11_, we found that compounds with *tert*-butyl groups in the terminal benzene ring of part Y_B_ exhibited higher cytotoxicity than those with EWGs. Interestingly, compound C_2_ (WF-208) exhibited stronger cytotoxicity than the other compounds A_1_–A_4_ and B_1_–B_12_. These findings prompt further studies on WF-208 (C2) mechanisms of action.

### Relief of zinc-mediated inhibition of procaspase-3 by WF-208

**Figure 2:**
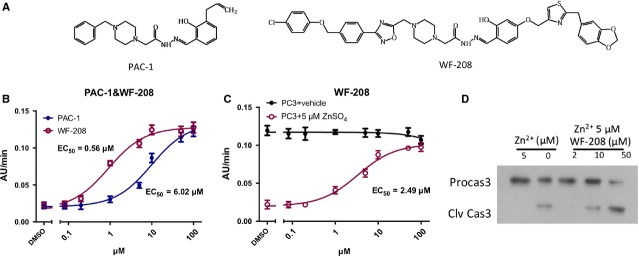
PAC-1 and WF-208 activated procaspase-3 *in vitro*. (A) Structures of PAC-1 and WF-208. (B) The PAC-1 and WF-208 activated rh-procaspase-3 in series of concentration 0.08–100 μM. (C) PAC-1 and WF-208 activated rh-procaspase-3 in zinc free vehicle and 5 μM ZnSO_4_ added vehicle. (D) The cleavage of procaspase-3 induced by WF-208 in zinc-free and 5 μM ZnSO_4_ buffer (PC3: procaspase-3, C3: caspase-3).

As shown in Figure2, the concentration-dependent activity of both WF-208- and PAC-1-induced procaspase-3 activation followed typical concentration-response curve *in vitro*. WF-208 significantly activated procaspase-3 (EC_50_ = 0.56 μM), which was more efficacious than PAC-1 (EC_50_ = 6.02 μM) (Fig.2B). Moreover, since previous studies have indicated that PAC-1 activates procaspase-3 through the inhibition of zinc chelation 14,23. In this study, the role of zinc in WF-208-induced procaspase-3 activation was also investigated. As shown in Figure2C, procaspase-3 in buffer without Zn^2+^ had high auto-activation. WF-208 activated procaspase-3 in the buffer containing 5 μM ZnSO_4_ rather than in the buffer without Zn^2+^. The Western blot results also revealed that zinc inhibits procaspase-3 auto-activation, and that WF-208 is able to revert this inhibition in a concentration-dependent manner.

### Cell death induction in cancer and normal cells by WF-208

**Figure 3:**
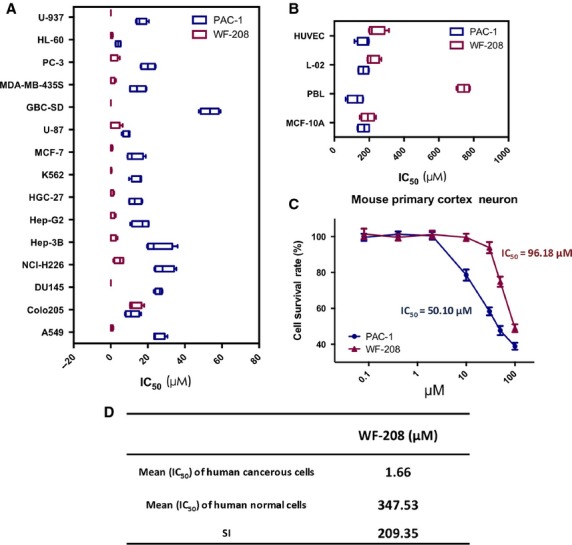
Cytotoxicity of PAC-1 and WF-208 against cancerous cells and normal cells. (A) The IC_50_ of PAC-1 and WF-208 in various cancerous cells. (B) The IC_50_ of PAC-1 and WF-208 in various normal cells. (C) The cytotoxicity of WF-208 and PAC-1 against mouse primary cortical neuron cells. (D) The mean of IC_50_ in cancerous cells and normal cells and selection index (SI) of WF-208.

The cytotoxicity of WF-208 was measured with MTT method using the human malignant cell lines (leukaemia, lung cancer, hepatoma, colon carcinoma, prostate carcinoma, breast carcinoma, gastric cancer, breast cancer, glioma, gallbladder carcinoma) and normal human cells after the treatment with different concentrations of WF-208 for 72 hrs. WF-208 displayed more cytotoxicity against tumour cells than PAC-1, with IC_50_ values ranging from 0.08 to 12.85 μM (Fig.3A and B). The overall mean IC_50_ value of WF 208 in the fifteen malignant cell lines was 1.66 μM (Fig.3C), which was an approximate 20-fold increase compared with PAC-1. In contrast, the sensitivity of the human primary cells and the human normal cell lines to WF-208 were much lower. The IC_50_ value for WF-208 on PBL is 742.62 μM, which was much higher than that for PAC-1. The mean IC_50_ value for WF-208 on three different normal cells was 215.79 μM, which was significantly higher than that for PAC-1 (*P* = 0.022). In addition, because of previously reported neurotoxicity of PAC-1 *in vitro* and *in vivo* 15,17, the neurotoxicity of WF-208 was also investigated. In this study, the cytotoxicity of WF-208 (IC_50_ = 96.18 μM) in mouse primary cortical cell was lower than PAC-1 (IC_50_ = 50.10 μM; Fig.3D). In summary, these results suggest a higher selective cytotoxicity against human malignant cells of WF-208.

### Cell apoptosis induction *via* activation of caspases-3 by WF-208

**Figure 4:**
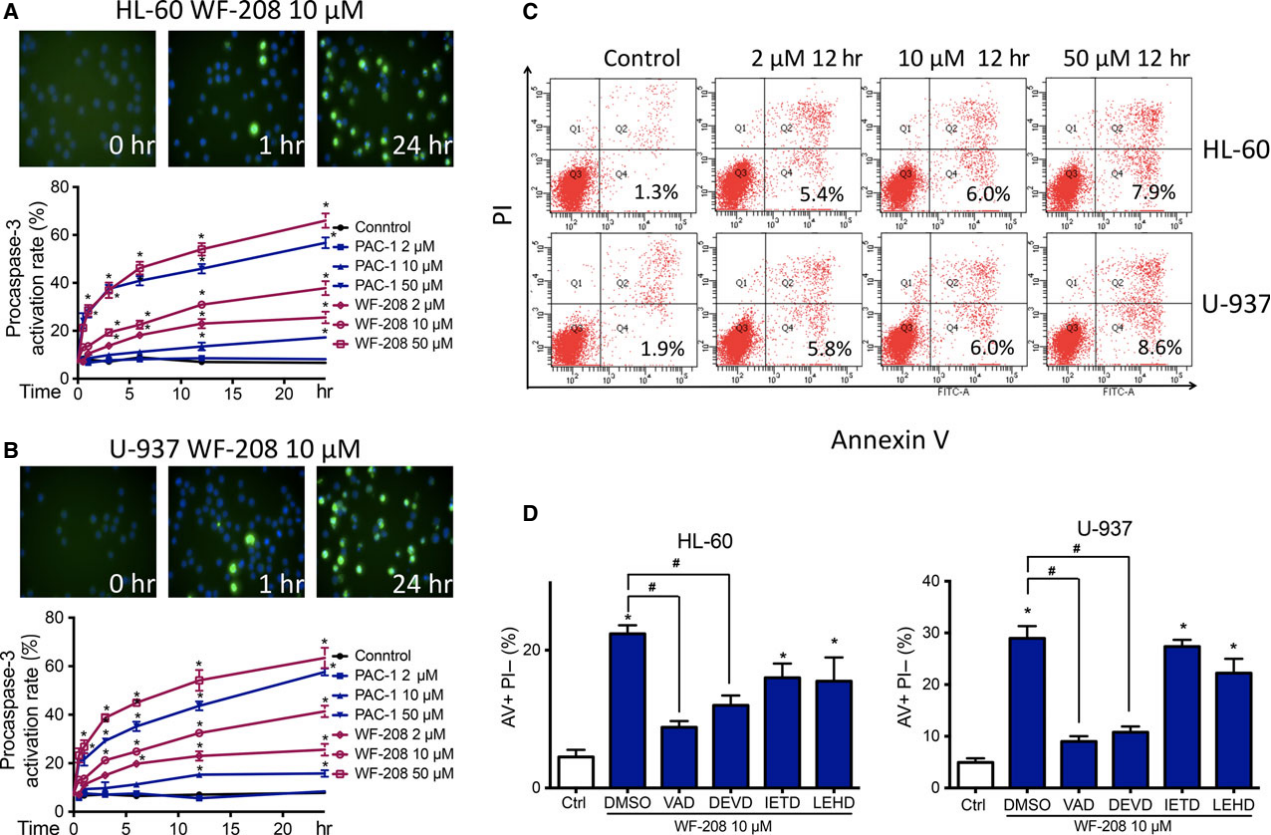
WF-208 induced HL-60 and U-937 apoptosis through the activation of procaspase-3 to caspase-3. (A) WF-208 and PAC-1 induced the cleavage of procaspase- 3 to caspase-3 in HL-60 cells (upon) and U-937 (below) at different time and concentration. *P < 0.05 *versus* control group. (B) WF-208 and PAC-1 induced the cleavage of procaspase- 3 to caspase-3 in U-937 cells. The representative image of WF-208 (upon) and activation rate of WF-208 and PAC-1 (below) induced procaspase-3 cleavage in U-937 cells at different time and concentration. *P < 0.05 *versus* control group. (C) Phosphatidylserine exposure (measured by Annexin V/PI co-staining, describe as the percentage of Annexin V-positive PI-negative cell percentage) in HL-60 and U-937 after 24 h treatment with 2, 10, 50 μM WF-208. (D) The inhibition of 50 μM vancaspase inhibitor (Z-VAD-FMK), caspase-3 inhibitor (Z-DEVD-FMK), caspase-8 inhibitor (Z-LETD-FMK), caspase-9 inhibitor (Z-LEHD-FMK) on early apopsotis induced by 10 μM WF-208 in HL-60 and U-937 cell lines treatment 24 hrs. *P < 0.05 *versus* control, ^#^*P* < 0.05 *versus*WF-208 alone.

Caspase-3 is one of the most crucial proteins of apoptosis, which exists as zymogen procaspase-3 before activation. After protein cleavage, procaspase-3 transform into its active form, caspase-3, followed by translocate from cytoplasm to nuclei 3. As shown in Figure4, immunofluoresence staining for cleaved caspase-3 was detected by high content analysis system. Nucleus was stained with Hoechst 33342. After treatment with 10 μM of WF-208 for 1 hr, cleaved caspase-3 was observed. After treatment for 24 hrs, procaspase-3 was cleaved in more than 60% cells (Fig.4A and B). At the same concentrations, the apoptosis per cent of WF-208 group was significant higher than which in PAC-1 group (Fig.4A and B). Chromatin condensation was also apparent in Hoechst-33342-stained HL-60 and U-937 treated with WF-208. These results indicate that WF-208 could activate procaspase-3 in time- and concentration-dependent manner in culture cells.

**Figure 5:**
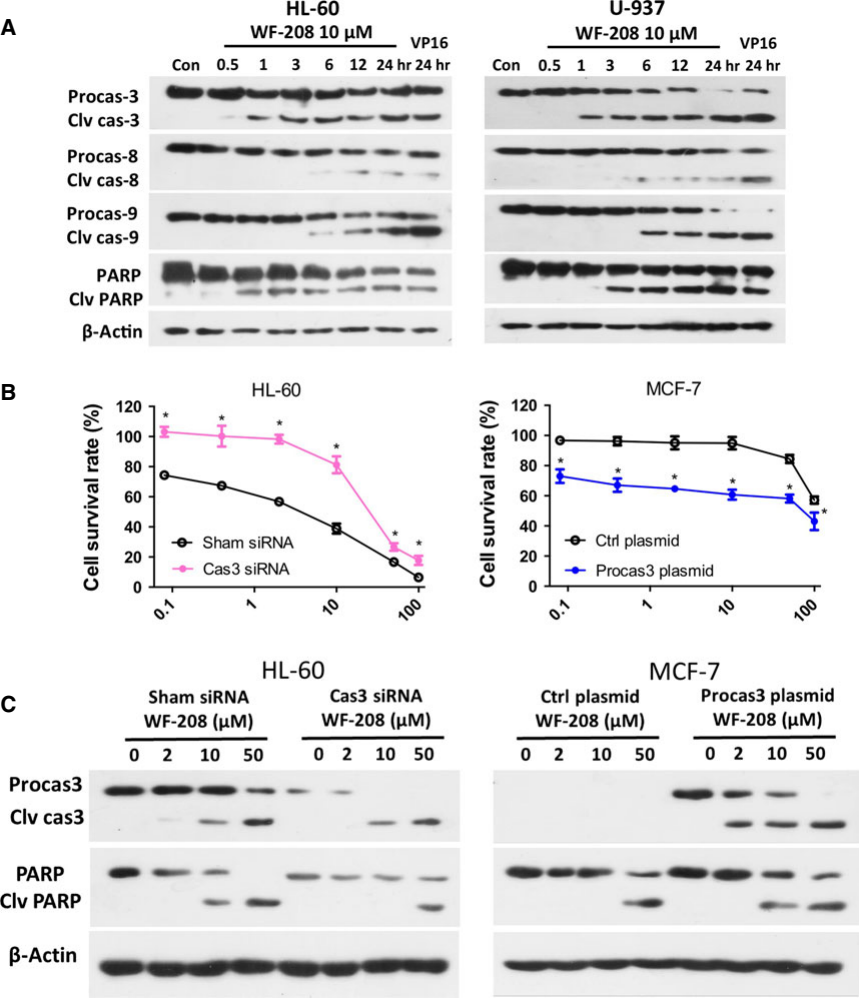
The WF-208-indeced apoptosis was caspase-3-dependent. (A) The effects of WF-208 on caspase family proteins and PARP at different time were measured by Western blot analyses in HL-60 (left) and U-937 (right) cells. (B) The cytotoxicity of WF-208 on procaspase-3 silenced HL-60 cells (left) and procaspase-3 overexpressed MCF-7 cells (right) after 24 hrs treatment. *P < 0.05 compare with sham siRNA or control plasmid group. (C) The WF-208-induced cleavage of caspase-3 and PARP in procaspase-3 silenced HL-60 cells (left) and procaspase-3 overexpressed MCF-7 cells (right).

The apoptosis rates of procaspase-3 overexpressed HL-60 and U-937 cells treated with WF-208 were measured by Annexin-V-FITC/PI co-stain assay. As shown in Figure5C, WF-208 caused considerable phosphatidylserine exposure on the outer leaflet of the cell membrane. This phenomenon was observed in a concentration-dependent manner.

To investigate whether caspases were involved in WF-208-induced apoptosis, the inhibitors of vancaspase, caspase-3, caspase-8, caspase-9 were pre-treated with WF-208. The vancaspase inhibitor (Z-VAD-FMK) and caspase-3 inhibitor (Z-DEVD-FMK) had comparable ability to abolish the WF-208-induced early apoptosis. Interestingly, the caspase-8 inhibitor (Z-IEDT-FMK) and the caspase-9 inhibitor (Z-LEHD-FMK) could partly inhibit WF-208-induced apoptosis, with the inhibition significantly lower than Z-VAD-FMK or Z-DEVD-FMK (Fig.5D). These observations suggest that different caspases may be involved in WF-208 induced apoptosis, especially caspase-3.

Procaspase-3 was over-expressed in HL-60 and mutated in MCF-7. In this study, we proposed to test whether WF-208 was able to induce apoptosis in HL-60 cells silenced procaspase-3 or MCF-7 cells transfected with procaspase-3. Overexpressed procaspase-3 in HL60 cells was silenced with procaspase-3 siRNA. The silencing efficiency was more than 60%, using scrambled siRNA as control. After the treatment with WF-208 at different concentrations for 24 hrs, the cytotoxic effect of WF-208 in procaspase-3-silenced HL-60 cells was less than control HL-60 cells (Fig.5B). Furthermore, the cleaved PARP induced by WF-208 in procaspase-3-silenced HL-60 cells was also less than that in control HL-60 cells (Fig.5C). MCF-7 cells was transiently transfected with the pIRES2-EGFP-procaspase-3 plasmid to expression procaspase-3, using empty vector as control. After the treatment with WF-208 at different concentrations, the cytotoxic effect of WF-208 in MCF-7 cells transfected with procaspase-3 was more sensitive than that of MCF-7 cells transfected with empty vector (Fig.5B). Furthermore, the PARP cleaved induced by WF-208 in MCF-7 cells transfected with procaspase-3 was also more obvious than that of MCF-7 transfected with empty vector (Fig.5C). These interesting results suggest that WF-208-induced cancer cells apoptosis may be caspase-3-dependent, although a contribution of caspase-3 independent pathways cannot be excluded.

### WF-208 induced changes in the expression of IAPs through proteasome in HL-60 and U-937 cells

**Figure 6:**
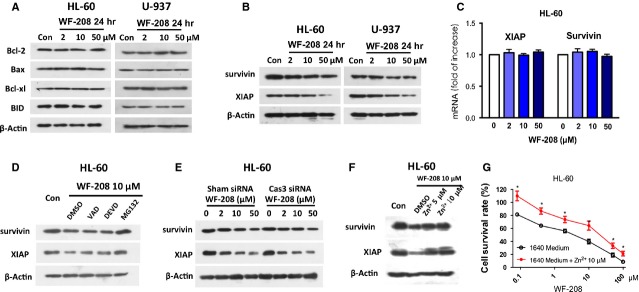
WF-208 decreases IAPs by promoting its degradation by the proteasome. (A) The effects of WF-208 on Bcl-2 family proteins at different concentration after 24 hrs were measured by Western blot analyses in HL-60 and U-937 cell lines. (B) The effects of WF-208 on XIAP and Survivin at different concentration after 24 hrs were measured by Western blot analyses in HL-60 and U-937 cell lines. (C) The effects of WF-208 on XIAP and survivin mRNA at different concentration measured by real-time PCR. (D) The effects of WF-208 combined with Ac-DEVD-FMK, Ac-VAD-FMK and MG132 on XIAP and survivin in 10 μM at 24 hrs. (E) The effects of WF-208 at different concentration after treatment 24 hrs of treatment on XIAP and survivin in normal HL-60 cells and procaspase-3 silenced HL-60 cells. (F) The effects of 5, 10 μM ZnSO_4_ co-treatment with WF-208 on XIAP and survivin in HL-60 after 24 hrs. (G) The cytotoxicity of WF-208 co-treatment with 10 μM ZnSO_4_ against HL-60 after 24 hrs.

To investigate the effects of WF-208 on other pro-apoptotic and anti-apoptotic proteins, the Bcl-2 family proteins (Fig.6A) and XIAP family proteins (Fig.6B) were detected. After treatment of various concentrations of WF-208 for 24 hrs, the expression of Bcl-2 family members, Bcl-2, Bcl-xl, Bax and BID, was not changed (Fig.6A). However, the expression of IAP family XIAP and survivin were significantly down-regulated by WF-208 in a concentration-depended manner (Fig.6B). These data suggest that IAP family members, but not Bcl-2 family members, are likely to play an important role in WF-208-induced apoptosis of malignant tumour cells.

To further determine how XIAP and survivin were involved in the WF-208-induced apoptosis, a series of experiments were designed. First, previous reports indicate that activated caspase-3 could degrade XIAP, so the caspase-3 inhibitor or vancaspase inhibitor was pre-treatment with WF-208 separately. As shown in Figure6D, WF-208 10 μM treatment for 24 hrs could decrease XIAP and survivin. Neither caspase-3 inhibitor nor vancaspase inhibitor could increase the level of XIAP and survivin compared to WF-208 treatment only. Meanwhile, WF-208 decreased XIAP and survivin in a concentration manner in both procaspase-3 silenced HL-60 cells and control HL-60 cells (Fig.6E). The degree of WF-208-induced XIAP and survivin down-regulation in HL-60 had no marked change after procaspase-3 silenced. Therefore, WF-208-induced XIAP and survivin down-regulation may not be through the activation of procaspase-3. Second, real-time PCR was used to measure levels of XIAP and survivin mRNA after WF-208 treatment. As shown in Figure6C, WF-208 failed to change the XIAP and survivin mRNA. Third, because the degradation of XIAP and survivin was mainly through proteasome 24, a proteasome inhibitor MG132 was co-treated with WF-208. As shown in Figure6D, 5 μM of MG132 blocked the down-regulation of XIAP and survivin induced by WF-208. Finally, zinc-chelating agent TPEN degraded XIAP in prostatic carcinoma cells 24. So, zinc was co-treated with WF-208. As shown in Figure6F, 5 and 10 μM ZnSO_4_ blocked the down-regulation of XIAP and survivin induced by WF-208. We also detected the cytotoxicity of WF-208 co-treatment with 10 μM ZnSO_4_. As shown in Figure6G, 10 μM ZnSO_4_ significantly relieved the cytotoxicity of WF-208. This effect may be related to the procaspase-3 activation and IAPs down-regulation effect of WF-208. In summary, the possible mechanisms of WF-208 down-regulating XIAP and survivin were through chelating zinc and increasing proteasome-related degradation but not caspase-3- related degradation.

### WF-208 activated procaspase-3, reduced IAPs and inhibited tumour growth in prostate carcinoma xenograft tumour models

**Figure 7:**
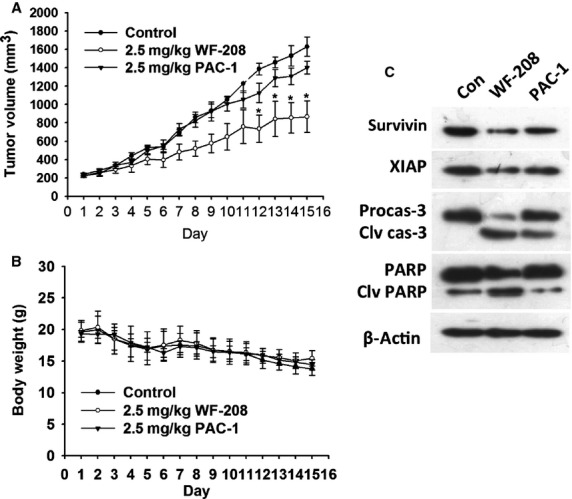
WF-208 inhibited tumour growth *in vivo*. (A) The tumour volume of PC-3 xenograft nude mice after 2.5 mg/kg WF-208 or PAC-1 i.v. exposure for 15 days. (B) The body weight of PC-3 xenograft nude mice after 2.5 mg/kg WF-208 and PAC-1 i.v. exposure for 15 days. (C) The protein level of caspase-3, PARP, XIAP and survivin proteins in tumour tissues of PC-3 xenograft nude mouse after drug exposure. *p < 0.05 compare with control group.

To evaluate the effect of WF-208 on the growth of malignant tumours *in vivo*, we examined the ability of WF-208 to suppress tumour growth in mouse PC-3 xenograft models. After 15 days treatment with WF-208 (2.5 mg/kg i.v.), the tumour volume of PC3-induced xenograft tumours was significantly reduced compared with control group. But there is no significantly difference of tumour volume between PAC-1 (2.5 mg/kg i.v.) group and control group (Fig.7A). Both WF-208 and PAC-1 had minimal influence on bw of nude mice (Fig.7B). Interestingly, the expression of activated caspase-3 and cleaved PARP was increased, while the expression of survivin and XIAP was decreased, suggested that the antitumour effects *in vivo* of WF-208 may be related to activation of procaspase-3 and down-regulation of IAPs (Fig.7C). These results were consistent with those of HL-60 and U-937 cells *in vitro*.

## Discussion

In present work, we showed a series of novel PAC-1 derivatives, which reduced cell viability of HL-60 cells. Among them, C2 (WF-208) was standout since its promising activity. WF-208 could induce cell death through activation of procaspase-3 and degradation of IAPs. The mechanisms of these effects may be related with chelation of zinc.

HL-60, which is overexpressed procaspase-3 13, was used to evaluate the cytotoxic activities of novel PAC-1 derivatives. Most of derivatives exhibited more potent activities against HL-60 cells compared to PAC-1. Especially, compound C_2_ (WF-208) showed promising activity against HL-60 with the IC_50_ value of 0.08 μM, which was 26.3-fold lower than PAC-1. From the preliminary SAR, we may conclude that the novel compounds with EWGs on phenyl X showed better antitumour effect *in vitro* than others. Furthermore, compared to compounds with EWGs on Y terminal benzene ring, compounds B_4_ and B_10_ with electron-donating group, such as tert-butyl groups, which located at para position, led to a significant improvement in cytotoxic activity. Overall, the SAR of these compounds herein paves the way for the development of more potent versions of procaspase-3 activators.

Procaspase-3 activating compounds, such as PAC-1, hold promise as novel therapeutics for the treatment of cancer. Several compounds of PAC-1 class have been discovered in the past 10 years 13,23,25,26. Compared with PAC-1, WF-208 has at least twenty times greater activity in variety of culture cancer cells, with lower toxicity in normal cells and lower neurocytotoxicity. Other PAC-1 derivatives, such S-PAC-1 16, ZnA-DPA, ZnA-Pyr 25 and six PAC-1 derivatives 26, were found to be only two or four times greater activity *in vitro* than PAC-1. In addition, WF-210, another procaspase-3 activator we reported previously, has similar activity in variety of culture cancer cells and portent anticancer effect in xenograft models. Thus, WF-208 is one of derivatives we discovered, which provide more efficacy and less toxicity than other procaspase-3 activators reported before.

Many anticancer drugs induce cancer cell death through apoptosis 27. Activation of caspase is the central step of apoptosis, which caspase-3 is an important one 2. Our study proved WF-208 induced apoptosis through activation of procaspase-3 in many evidence. Although many anticancer drugs kill cancer cells through apoptosis, our pervious study indicated procaspase-3 did not play an indispensable in etoposide, MG132, staurosporine and Fas Ligand- induced apoptosis. In consideration of pervious study has been reported caspase-3 mediated feedback activation of apical caspase 28, the WF-208 induced activation of procaspase-8 and procaspase-9 may be caspase-3-dependent.

Compounds in the PAC-1 class activate procaspase-3 *via* chelation of inhibitory zinc ions 14,26. Other zinc chelators such as TPEN 25 that give caspase-3 activation and apoptosis have been reported. Intercellular zinc is found principally in tightly bound complexes in metalloproteinases, zinc finger domains and other metal binding proteins; however, ∼10% of cellular exist in a loosely bound labile pool 29. Chelation of loosely bound pool of zinc could activate caspase-3 rapidly and directly without a requirement for an upstream event 13,14,30. Thus, the evaluation of WF-208 is a proof of concept for the chelation of loosely bound pool of zinc as an anticancer therapy.

By inhibiting caspases-3, -7 and -9, IAPs block the convergence point of multiple caspase activation pathways and thus inhibit apoptosis from multiple stimuli 31. The realization that alterations in IAP proteins are found in many types of human cancer and are associated with chemoresistance, disease progression and poor prognosis. XIAP and survivin are the IAP family proteins that have evolved to potently inhibit the enzymatic activity of mammalian caspase and promote resistance to apoptosis 32. In this study, we found WF-210 could decrease XIAP and survivin, and zinc could block this effect. In the previous study, zinc chelator TPEN showed XIAP-degradation activity 24, indicating that except directly influence on caspase-3, zinc also shows cytoprotective function by stabilizes XIAP in apoptosis pathway. In the preclinical study, IAP antagonists such as Smac mimetic sensitize the pro-apoptotic signalling and effect in combination with other cytotoxic agents. WF-208 not only active procaspase-3 but also degrade XIAP and survivin 33. The therapeutic potential of WF-208 might be more abroad than single-target drugs targeting procaspase-3 or IAPs.

In conclusion, a series of PAC-1 derivatives were firstly synthesized and screened their anticancer activity. Among these derivatives, WF-208 is a potential anticancer chemical, which displayed enhanced procaspase-3 activating ability and potent cytotoxicity for cancer cells but had no significant toxicity for normal cells. This study may provide valuable insight for future design and development of antitumour agents with potent activities.
